# Validated repeatability of patient-reported outcome measures following primary total hip replacement: a mode of delivery comparison study with randomized sequencing

**DOI:** 10.1080/17453674.2018.1521183

**Published:** 2018-11-19

**Authors:** Charlotte V E Carpenter, Julia Blackburn, John Jackson, Ashley W Blom, Adrian Sayers, Michael R Whitehouse

**Affiliations:** 1 Musculoskeletal Research Unit, Bristol Medical School, 1st Floor Learning & Research Building, Southmead Hospital, Bristol;; 2 Department of Trauma and Orthopaedics, Bristol Royal Infirmary, Upper Maudlin St, Bristol;; 3 Department of Trauma and Orthopaedics, North Devon District Hospital, Raleigh Park, Barnstaple, UK

## Abstract

Background and purpose — Patient-reported outcome measures (PROMs) are used to understand better the outcomes after total hip replacement (THR). These are administered in different settings using a variety of methods. We investigated whether the mode of delivery of commonly used PROMs affects the reported scores, 1 year after THR.

Patients and methods — A prospective test–retest mode comparison study with randomized sequence was done in 66 patients who had undergone primary THR. PROMs were administered by 4 modes: self-administration, face-to-face interview, telephone interview, and postal questionnaire. PROMs included: Western Ontario and McMaster Universities Osteoarthritis Index (WOMAC), Oxford Hip Score (OHS), EQ5D-3L (EQ5D), and Self-Administered Patient Satisfaction Scale (SAPS). Linear regression was used to estimate relationships between the mean scores for PROMs by mode. Individual paired differences by mode were calculated, relationships between modes were identified, and results adjusted by time delay and participant age.

Results — There was no statistically significant difference between the mean PROM scores recorded for each mode of delivery for each score. Statistically significant differences in the individual paired differences were detected between modes for the WOMAC stiffness subscale, OHS, EQ5D, and SAPS. OHS difference in individual paired means between face-to-face and telephone interview exceeded the minimal clinically important difference.

Interpretation — PROMs mode of administration can affect the recorded results. Modes should not be mixed and may not be comparable between studies. It should not be assumed that different modes will obtain the same results and where not already established this should be checked by researchers before use.

There are various metrics used to judge the success or failure of total hip replacement (THR). Hard endpoints such as revision of the THR and mortality are popular as they are easy to define, but such outcomes fail to take account of the degree of relief of symptoms experienced by the patient, i.e., soft endpoints (Wylde and Blom 2011). To better understand the outcomes after THR, patient-reported outcome measures (PROMs) have been widely adopted, and typically these PROMs are focused around domains such as pain, function, and stiffness. Their use has become routine and widespread, for example, the UK Department of Health’s National PROMs program (http://www.hscic.gov.uk/proms) administers PROMs prior to and 6 months after intervention for procedures such as THR, total knee replacement, hernia repair, and varicose vein surgery.

PROMs questionnaires are administered and completed in a number of different settings using a variety of methods (Tourangeau et al. 2000). Common modes of delivery include paper based, face-to-face, telephone, and computer delivered, with responses being self-recorded or assisted by a third party. With the evolution of technology, the boundaries between modes of delivery are now becoming blurred. In addition, preoperative and postoperative assessments are frequently performed using different modes of administration, and in many research studies a mixture of modes is used to ensure data completeness (Dillman et al. 2009).

When modes of delivery are mixed, it is important to understand whether the mode of delivery of the questionnaire affects the psychometric properties of the score (Honaker 1988). If different modes of delivery result in scores that are not equivalent, then these modes should not be mixed in a single study design. Factors that may be associated with the magnitude of difference include context, content, and the population studied (Hood et al. 2012). Systematic review of mode comparisons has shown that modes are vulnerable to bias when comparison is made between an interviewer being involved and self-completion (Hood et al. 2012). In a large population study, telephone administration yielded more positive health-related quality of life estimates than self-administration (Hanmer et al. 2007). However, multiple item scales are less prone to bias and differences between modes have ameliorated as technologies such as telephone- and computer-based completion have become commonplace (Gwaltney et al. 2008).

We investigated whether the mode of questionnaire delivery influences test scores in commonly used PROMS in primary THR.

*Study design* — Patients were invited to participate in a test–retest study of 4 PROMs using 4 modes of delivery, 1 year following THR, using a randomized crossover design.

## Patients and methods

A prospective mode comparison cohort study was conducted in a single NHS tertiary orthopedic center. In order to ensure that patients had reached a steady state in terms of their outcome following surgery, patients who were 1 year following THR were invited to participate (Lenguerrand et al. 2016).

Patients were eligible for inclusion in the study if they had undergone primary THR for any indication 1 year previously. Exclusion criteria were patients who had undergone revision THR, patients who were unwilling or unable to provide informed consent, and patients who were unable to understand or complete questionnaires in English (Study flow chart, Figure).

Patients were recruited to the study by written invitation sent 1 week before their outpatient clinic appointment. Recruitment occurred between June 2014 and October 2015. 66 patients, who indicated they wished to participate, were consented and randomized to the order in which they would receive the questionnaires. Participants were asked to complete a set of 4 questionnaires: the Western Ontario and McMaster Universities Osteoarthritis Index (WOMAC); the EQ5D-3L health questionnaire (EQ5D); the Self-Administered Patient Satisfaction Scale (SAPS); and the Oxford Hip Score (OHS). 4 modes of questionnaire completion were used: self-administered in clinic; face-to-face interview in clinic; telephone interview; and postal questionnaire. For full details of PROMS questionnaires, questions, and structure of questionnaires please see Appendix.

The sets of PROMs were delivered by 4 modes: self-administered in clinic and face-to-face interviewer led, both completed during the outpatient clinic appointment; and later via telephone interview and self-administered by post. Participants completing PROMs self-administered in clinic were asked to complete the set of questionnaires using pen and paper, without assistance. During the face-to-face interview, each question in the set was asked by a member of the research team and the questionnaires were completed by the researcher based on the verbal responses of the participant.

### Randomization

The participants were randomized to the sequence in which mode of completion was done (Table 1). Participants were randomized in permuted blocks to 1 of 4 groups with 3 times block size.

**Table 1.** Randomization sequence

Group 1	Group 2	Group 3	Group 4
Time point 1 in clinic			
1. Self-administered	1. Face-to-face	1. Self-administered	1. Face-to-face
2. Face-to-face	2. Self-administered	2. Face-to-face	2. Self-administered
Time point 2			
Telephone interview	Postal	Postal	Telephone interview
Time point 3			
Postal	Telephone interview	Telephone interview	Postal

### Statistics

#### Sample size

A sample size of 60 was calculated, based on the OHS. The minimal clinically important difference (MCID) for the OHS is 5 (Beard et al. 2015). Therefore, for 80% power and a 2-sided 5% significance, a sample size of 52 is required to allow the study to detect if there is a significant difference between the scores by different modes. This was rounded up to 60 participants to account for loss to follow-up.

#### Missing values

Missing values were identified before statistical analysis. For calculation of the PROM scores, missing values were dealt with according to the user guides for each score. For WOMAC, if 2 or more pain, both stiffness, or 4 or more physical function items were omitted, the participants’ responses were deemed invalid and the deficient subscale was not used for analysis. According to OHS guidance, if more than 2 questions were unanswered, a score was not calculated. If 1 or 2 questions were unanswered, the mean value of other responses was substituted for the missing value. An EQ5D index was calculated from the EQ5D-3L scores. EQ5D questionnaires with missing values were excluded. There is no specific way of dealing with missing data for SAPS; however, there were no missing values.

#### Analysis

There are 2 possible methods to analyze mode comparison studies. The first is to assume independence on each occasion a questionnaire is completed. This analytical standpoint is typically described as investigation of between-population differences. In this case, linear regression was used to estimate the between-population mean difference of each questionnaire by delivery mode of delivery. The second method of analysis investigates within-individual (paired) differences of each questionnaire by mode of delivery. In this case, the paired difference between each mode was calculated and linear regression was used to estimate any within individual differences between modes of delivery. Within-patient analyses were further adjusted for the time between completion of questionnaires (time delay) and age of the participant, i.e., < 70 or ≥70 years and old. P-values are reported without adjustment for multiple testing (Perneger 1998). Data were analyzed using STATA (version 14, StataCorp, College Station, TX, USA).

### Ethics, registration, funding, and potential conflicts of interest

Ethical approval was granted for the study by the West of Scotland Research Ethics Service on April 24, 2014 (Ref: 14/WS/0062). This study was supported by the NIHR Biomedical Research Centre at the University Hospitals Bristol NHS Foundation Trust and the University of Bristol. The views expressed in this publication are those of the author(s) and not necessarily those of the NHS, the National Institute for Health Research, or the Department of Health. Adrian Sayers is funded by an MRC Strategic Skills Fellowship (MR/L01226X/). No competing interests are declared.

## Results

66 participants (median age 69 (IQR 62–77), 33 male) consented to participate (Figure).

### Missing data

Missing data were observed for each of the WOMAC, OHS, and EQ5D measures. There were no incomplete SAPS questionnaires. More missing data were observed for the OHS than for any of the other scores (Table 2, see Supplementary data). This resulted in the exclusion of 5 OHS questionnaires, 3 of which were when the questionnaire was self-administered in clinic. In comparison, the WOMAC score was insufficiently completed for the inclusion of all 3 subscales in 3 cases and 1 EQ5D questionnaire was excluded. Scores were left unanswered most frequently when self-administered in clinic (29 of 2,970 data points).

**Table 2.** Missing data. Values are frequency

	Face-to-face			Self-administered			Telephone			Postal		
	Pts	Items	Excluded	Pts	Items	Excluded	Items	Pts	Excluded	Pts	Items	Excluded
WOMAC stiffness	0	0	0	0	0	0	1	1	0	1	2	1
WOMAC pain	0	0	0	0	0	0	0	0	0	0	0	0
WOMAC function	2	6	1	2	6	1	4	5	0	3	6	0
OHS	2	13	1	4	23	3	1	1	0	1	12	1
EQ5D	0	0	0	0	0	0	0	0	0	1	1	1
SAPS	0	0	0	0	0	0	0	0	0	0	0	0

Pts: participants

**Table:** Table 2. Continued

	SI—P				SI—T				P—T			
Model	dif.	SE	95% CI	p-value	dif.	SE	95%CI	p-value	dif.	SE	95% CI	p-value
WOMAC Stiffness												
1	0.60	2.1	(–3.6 to 4.8)	0.8	5.95	1.8	(2.3 to 9.6)	0.002	4.62	1.5	(1.7 to 7.6)	0.003
2	–0.83	1.8	(–4.3 to 2.7)	0.6	5.28	1.6	(2.0 to 8.6)	0.002	4.94	1.6	(1.8 to 8.1)	0.003
3	–4.19	2.1	(–8.4 to 0.001)	0.05	5.87	2.5	(0.9–11)	0.002	3.04	2.1	(–1.1 to 7.2)	0.1
WOMAC Pain												
1	1.58	1.0	(–0.36 to 3.5)	0.1	1.62	1.2	(–0.73 to 4.0)	0.2	0.71	0.8	(–0.91 to 2.3)	0.4
2	1.31	1.0	(–0.66 to 3.3)	0.2	1.78	1.3	(–0.77 to 4.3)	0.2	0.42	0.8	(–1.2 to 2.0)	0.6
3	0.45	1.2	(–2.0 to 3.0)	0.7	1.66	2.0	(–2.3 to 5.6)	0.4	0.30	1.1	(–1.9 to 2.5)	0.8
WOMAC Function												
1	0.81	1.6	(–2.4 to 4.1)	0.6	3.15	1.5	(0.21 to 6.1)	0.04	2.61	1.3	(0.11 to 5.1)	0.04
2	–0.07	1.4	(–3.0 to 2.8)	1	2.69	1.4	(–0.06 to 5.4)	0.06	2.62	1.3	(–0.02 to 5.3)	0.05
3	–0.24	1.8	(–3.9 to 3.4)	0.9	2.72	2.1	(–1.5 to 6.9)	0.2	2.11	1.8	(–1.1 to 5.6)	0.2
OHS												
1	0.96	1.0	(–1.1 to 3.0)	0.4	–0.69	1.0	(–2.6 to 1.3)	0.5	–1.71	0.44	(–2.6 to –0.83)	<0.001
2	1.06	1.0	(–1.0 to 3.1)	0.2	–0.63	1.0	(–2.8 to 1.5)	0.6	–1.81	0.46	(–2.7 to –0.88)	<0.001
3	0.96	1.3	(–1.6 to 3.6)	0.5	–0.29	1.7	(–3.6 to 3.1)	0.9	–1.24	0.59	(–2.4 to –0.07)	0.04
EQ5D index												
1	–0.01	0.02	(–0.06 to 0.03)	0.6	–0.04	0.02	(–0.09 to 0.01)	0.1	–0.02	0.02	(–0.06 to 0.02)	0.5
2	–0.02	0.02	(–0.06 to 0.01)	<0.001	–0.05	0.03	(–0.10 to –0.001)	0.05	–0.02	0.02	(–0.07 to 0.02)	0.3
3	–0.02	0.03	(–0.07 to 0.03)	0.4	–0.05	0.04	(–0.13 to 0.03)	0.2	0.00	0.03	(–0.06 to 0.06)	1.0
SAPS												
1	–3.77	0.93	(–5.6 to –1.9)	<0.001	–4.90	0.95	(–6.8 to –3.0)	<0.001	–0.30	0.57	(–1.4 to 0.84)	0.6
2	–3.69	0.92	(–5.5 to –1.9)	0.002	–4.77	1.0	(–6.8 to –2.7)	<0.001	–0.21	0.60	(–1.4 to 1.0)	0.7
3	–4.10	1.2	(–6.4 to –1.8)	0.001	–5.28	1.5	(–8.3 to –2.2)	0.001	0.09	0.81	(–1.5 to 1.7)	0.9

Model 1 = Individual paired differences (IPD), Model 2 = IPD + time delay, Model 3= IPD + time delay + age.

F2F: face to face in clinic; SI: Self-administered in clinic; P: postal; T: telephone interview.

Dif: Individual paired difference: 95% CI: 95% confidence interval.

### Between-population mode differences

There were no statistically significant differences among mean scores by mode of delivery for any PROM investigated (Table 3, see Supplementary data).

**Table 3.** Mean differences by mode. Values are mean (standard deviation)

PROM	Face-to-face	Self-administered	Postal	Telephone	F test
WOMAC stiffness	15.9 (22)	15.5 (22)	13.3 (16)	9.1 (15)	P F (3, 259) = 0.2
WOMAC pain	12.1 (17)	12.1 (17)	10.2 (16)	10.1 (16)	P F (3, 260) = 0.8
WOMAC function	15.4 (19)	15.4 (19)	14.1 (18)	12.1 (16)	P F (3, 260) = 0.7
OHS	39.8 (10)	40.2 (10)	40.1 (9.8)	42.0 (7.7)	P F (3, 258) = 0.6
EQ5D index	0.74 (0.30)	0.74 (0.30)	0.77 (0.30)	0.77 (0.24)	P F (3, 259) = 0.8
SAPS	93.4 (8.7)	89.2 (16)	93.6 (14)	94.3 (13)	P F (3, 260) = 0.09

### Timing between administration

There was no difference between administrations 1 and 2, as these were undertaken on the same day, before and after an outpatient appointment. The median time difference between administration 2 and 3 was 7 (IQR 3–8) days and 6 (IQR 3–7) between timepoints 3 and 4. The median time between administrations 1 and 4 was 14 (IQR 8–17) days.

## Within-individual (paired) mode differences

### WOMAC subscales

When the WOMAC subscales were considered, there was a difference in the individual paired differences observed for the stiffness subscale between the modes of delivery (Table 4). This persisted when adjustment was made for age and the time delay between modes of delivery. Young patients were more likely to give a higher (worse) score when the score was completed in clinic by face-to-face interview or self-administered than when the score was completed by postal or telephone modes.

**Table 4.** Individual paired differences by mode

F2F—SI					F2F—P				F2F—T			
Model	dif.	SE	95% CI	p-value	dif.	SE	95%CI	p-value	dif.	SE	95% CI	p-value
WOMAC Stiffness												
1	0				0.60	2.1	(–3.6 to 4.8)	0.8	5.95	1.8	(2.3 to 9.6)	0.002
2	0				–0.83	1.8	(–4.3 to 2.7)	0.6	5.28	1.6	(2.0 to 8.6)	0.002
3	0				13.5	5.6	(2.2 to 25)	0.02	5.87	2.5	(0.9 to 11)	0.02
WOMAC Pain												
1	0				1.59	1.0	(–0.36 to 3.5)	0.1	1.61	1.0	(–0.35 to 3.5)	0.1
2	0				1.31	1.0	(–0.66 to 3.3)	0.2	1.78	1.3	(–0.77 to 4.3)	0.2
3	0				4.91	3.3	(–1.7 to 12)	0.1	1.66	2.0	(–2.3 to 5.6)	0.4
WOMAC Function												
1	0				0.81	1.6	(–2.4 to4.1)	0.6	3.15	1.5	(0.21 to 6.1)	0.04
2	0				–0.07	1.4	(–3.0 to 2.8)	1	2.69	1.4	(–0.06 to 5.4)	0.06
3	0				0.54	4.9	(–9.2 to 10)	0.9	2.72	2.1	(–1.5 to 6.9)	0.2
OHS												
1	–1.41	1.6	(–4.7 to 1.8)	0.4	–1.18	1.2	(–3.5 to 1.2)	0.3	–2.20	1.1	(–4.5 to 0.08)	0.06
2	–1.41	1.6	(–4.6 to 1.8)	0.4	–1.09	1.2	(–3.5 to 1.3)	0.4	–2.26	1.3	(–4.8 to 0.24)	0.08
3	–1.40	1.6	(–4.6 to 1.8)	0.4	5.34	3.9	(–2.5 to 13)	0.2	–7.35	1.7	(–11 to –4)	<0.001
EQ5D index												
1	0				–0.01	0.02	(–0.06 to 0.03)	0.6	–0.04	0.02	(–0.09 to 0.01)	0.1
2	0				–0.02	0.02	(–0.06 to 0.01)	0.2	–0.05	0.03	(–0.1 to –0.001)	0.05
3	0				–0.03	0.07	(–0.17 to 0.10)	0.6	–0.05	0.04	(–0.13 to 0.03)	0.2
SAPS												
1	4.29	1.8	(0.80 to 7.8)	0.02	0.20	1.4	(–2.6 to 3.0)	0.9	–0.48	1.4	(–3.2 – 2.3)	0.7
2	4.30	1.7	(0.80 to 7.8)	0.02	–0.10	1.4	(–2.9 to 2.7)	0.9	0.11	1.5	(–2.8 to 3.0)	0.9
3	4.26	17	(0.77 to 7.7)	0.02	–4.26	4.7	(–14 to 5.2)	0.4	–2.10	2.2	(–6.5 to 2.3)	0.4

Model 1 = Individual paired differences (IPD), Model 2 = IPD + time delay, Model 3= IPD + time delay + age.

F2F: face to face in clinic; SI: Self-administered in clinic; P: postal; T: telephone interview.

Dif: Individual paired difference: 95% CI: 95% confidence interval.

The WOMAC function subscale revealed a similar pattern with higher (worse) scores given when the score was completed in clinic by face-to-face interview or self-administered and when the form was self-administered and delivered by post compared with telephone interview, but this difference disappeared when adjusting for the time delay between modes and age.

### OHS

The individual paired differences between the OHS scores for different modes showed a statistically significant difference between postal and telephone scores when unadjusted and adjusted for time delay. However, this may not be clinically relevant as the difference is below the MCID of 5. When the OHS was adjusted for age and the time delay between modes of delivery, lower (worse) scores were given when the form was completed in clinic by face-to-face interview (–7.35, 95% CI –11 to –4) or self-administered by post (–1.24, 95% CI –2.4 to –0.07) compared with telephone interview completion.

### EQ5D

When the EQ5D was considered, no differences in the individual paired differences were seen. However, when adjusted for time delay between questionnaires, higher (better) scores were seen in the telephone and postal groups compared with the other modes of delivery. When adjusting for the age of the respondents, this difference did not persist. When age was categorized into young (< 70) or old (≥ 70), the difference persisted in the older group, suggesting older patients are more likely to report a higher (better) score for the EQ5D when completed by telephone interview or self-administered and delivered by post.

### SAPS

There was a statistically significant reduction in the individual paired differences for the SAPS between self-administration in clinic and face-to-face interview, and between self-administration in clinic and delivery by post or telephone interview. This difference persisted when adjusting for time delay between modes and age. Each point on the SAPS scale has a value of 6.25%. The difference between groups was roughly 4%, thus the effect was less than 1 point in 1 response, and of unlikely clinical significance.

## Discussion

We investigated whether the mode of delivery of commonly used PROMs affected the results reported by patients who had undergone primary total hip replacement.

There were no statistically significant between population differences among mean scores in any PROMs investigated.

We observed more missing data for the OHS than other PROMs. This included 3 OHS questionnaires that had no responses completed. The OHS was the last questionnaire in the series. The higher missing data count in this score may be due to survey fatigue, or perhaps, most simply, that patients omitted the last page of the questionnaire (Porter et al. 2004). Most missing data were seen in the self-administered in clinic group across all PROMs.

In a systematic review of mode of administration of surveys, Bowling (2005) described 13 sources of bias that may affect the results obtained by different modes. That study concluded that non-response is likely to be influenced by mode of administration, with a higher non-response reported in postal than face-to-face and postal than telephone, suggesting that premature termination is less likely in the presence of a motivating interviewer.

This finding is echoed by Wood and McLauchlan (2006) and by Fitzpatrick et al. (2000) in the response rates to the OHS, with highest responses achieved by face-to-face and self-administered questionnaires and lowest with postal responses. Both studies reported that question 6 of the OHS (“In the past 4 weeks, for how long have you been able to walk before pain from your hip becomes severe (with or without a stick)?”) was the one most frequently left unanswered.

WOMAC subscales did not reveal any statistically significant difference in mean scores across mode of delivery. When adjusted for time delay and the age of the participant, young patients showed a small propensity to worse scores on the WOMAC stiffness subscale when the score was completed in clinic by face-to-face interview or self-administered. This difference was between 5% and 13%, below the MCID of 25% and is therefore not likely to be clinically significant (Quintana et al. 2005). No statistically significant differences were seen in WOMAC pain scales by any mode of delivery. These findings are similar to those of Bellamy et al. (2002), which showed no difference between telephone and onsite administration for the WOMAC knee score and electronic versus paper surveys for patients with hip and knee OA in 2002.

OHS gave a higher (better) score for telephone than postal scores or face-to-face interviews, with a difference between face-to-face and telephone of 7 points, which is in excess of the MCID and therefore could be clinically significant (Murray et al. 2007). Older participants may give better scores for EQ5D if recorded by post or telephone interview. However, quantifying the magnitude is difficult with the EQ5D index. A small reduction in satisfaction was seen when SAPS was completed by self-administered in clinic when compared with face-to-face interview and telephone interviews and postal responses. These findings suggest a small propensity to better score responses in telephone questionnaires than other modes. Telephone interviews may be subject to biases including social desirability bias, yes-saying, and interviewer bias (Bowling 2005). Indeed, it has been shown that satisfaction may be more positive if surveys are presented aurally than visually (Dillman et al. 2009). Health-related quality of life scores have been shown to be consistent when mode of administration was the same but telephone administration of EQ5D yields more positive results than self-administered in an older group with both US and UK weighting (Hanmer et al. 2007). Hays et al. (2009) found that the maximum effect size between postal versus telephone administration of the EQ5D was 0.5. Wood and McLauchlan (2006) found no difference in mode of administration between postal delivery and interview of the OHS at 10-year follow-up after THR. The OHS showed only a small increase in mode effect average, 1.2, in telephone versus postal administration, which was not deemed clinically relevant (Messih et al. 2014). However, in a meta-analysis of mode of administration of PROMS, self-completion and assisted completion produced equivalent scores overall but results were influenced by the setting in which questionnaires were completed (Rutherford et al. 2016).

We found that mode of administration of PROMs 1 year after THR may have small effects on the results obtained. Participants in our study were randomized according to the order of modes; therefore, we do not believe practice effects are likely to explain these differences. Telephone and postal responses were collected following self-administration in clinic and face-to-face interview in clinic. As such, a time delay between these sets was introduced. This had an effect in some cases but was adjusted for within the analysis. Although participants were encouraged to complete the self-administered PROMs themselves, whether they received help or support from friends, family members, or carers was not documented. Interviewer-led PROMs were completed by several different researchers, which may introduce bias to the answers obtained. Comorbidity and the indication for THR were not investigated as part of this study. Our findings may be generalizable to patients who have undergone THR but not those awaiting THR and only apply to English versions of these PROMs.

We did not investigate electronic modes of delivery of these PROMs, such as via mobile phone, hand-held devices in clinic and automated telephone responses. In an increasingly digital age, these modes of delivery are increasingly used and the effects are as yet unknown in this population.

The small variations in responses to PROMs delivered by different modes of administration are not likely to have a significant impact on the results in smaller studies. However, in large longitudinal studies where the timing of questionnaires may vary, these small biases may induce statistically significant chance findings. To ensure bias is minimized in studies using PROMs assessment after THR, we recommend that modes of administration are, wherever possible, not mixed in a single study. If multiple modes are used it is important to distinguish between modes of administration, and avoiding mixing self-administered and interviewer-led PROMs may minimize the effects. When outcomes are collected by different modes of administrations this should be acknowledged, and care should be used when interpreting results. Whilst using different modes in the same study may be useful in minimizing missing data in clinical studies, it is important to recognize it is not a panacea, and the primary response is still missing.

## Supplementary data

Appendix and Tables 2 and 3 are available as supplementary data in the online version of this article, http://dx.doi.org/10.1080/17453674.2018.1521183

MRW: Conception and design of the study. JB and AS: Design and acquisition of the data. JB, and JJ: Acquisition of data. CC, and AS: Analysis of data. All authors interpreted data and wrote the report. CC, JB, AS, MW contributed equally to this work

The following people contributed to the paper through the acquisition of data: Christopher Woodrow, Harriet Mitchell, Sophie Stanger, Samantha Dixon, Nicolas Toosi, and Charlotte Howie.

*Acta* thanks Sarah Whitehouse and Nienke Wolterbeek for help with peer review of this study

## Supplementary Material

Supplemental Material

## Appendix

### PROMs questionnaires

The WOMAC is a 24-item questionnaire designed to measure pain, function, and stiffness in hips and knees. It uses a 5-point Likert-type scale from 0 to 4 for each question (giving a total scale of 0–96) with higher scores indicating worse outcomes. The minimal clinically important difference (MCID) for the WOMAC is 15 for stiffness, 23 for pain, and 19 for function (Quintana et al.^2005^). A percentage score for each subscale of the WOMAC was calculated, giving a score out of 100 for stiffness, pain, and function. EQ5D-3L is a standardized, non-disease-specific questionnaire for evaluating health-related quality of life in 5 dimensions, including mobility, self-care, usual activities, pain or discomfort, and anxiety or depression. The EQ5D index is calculated to provide a single value for health status, using the user written EQ5D Stata command in Stata. SAPS is a short questionnaire used to evaluate patient satisfaction with total hip and knee replacement. 4 items are scored on a 4-point Likert scale with responses from very dissatisfied to very satisfied. The scale score is the unweighted mean of the scores, with 100 being most satisfied and 25 least. The OHS is a 12-item questionnaire designed to measure changes in pain and function after total hip replacement surgery. The OHS is scored on a 0–48 scale, where 48 represents a good hip and 0 the worst (Murray et al. 2007). The MCID for the OHS is 5 (Beard et al.^2015^).

The sets of PROMs were delivered by 4 modes: self-administered in clinic and face-to-face interviewer led, both completed during the outpatient clinic appointment; and later via telephone interview and self-administered by post. Participants completing PROMs self-administered in clinic were asked to complete the set of questionnaires using pen and paper, without assistance. During the face-to-face interview, each question in the set was asked by a member of the research team and the questionnaires were completed by the researcher based on the verbal responses of the participant.

#### Western Ontario and McMaster Universities Osteo­arthritis Index

The following questions concern the amount of pain you have recently experienced in your hip. For each question, please tick the amount of **pain** you have experienced during the **PAST 4 WEEKS** due to the hip **that you had replaced**. Please (3) tick one column.

1. How much pain do you have **walking on a flat surface**?

None ^1^ Mild ^2^ Moderate ^3^ Severe ^4^ Extreme ^5^

2. How much pain do you have **going up or down stairs**?

None ^1^ Mild ^2^ Moderate ^3^ Severe ^4^ Extreme ^5^

3. How much pain do you have **at night while in bed**?

None ^1^ Mild ^2^ Moderate ^3^ Severe ^4^ Extreme ^5^

4. How much pain do you have **sitting or lying**?

None ^1^ Mild ^2^ Moderate ^3^ Severe ^4^ Extreme ^5^

5. How much pain do you have **standing upright**?

None ^1^ Mild ^2^ Moderate ^3^ Severe ^4^ Extreme ^5^

The following questions concern your physical function. By this we mean your ability to move around and look after yourself. For each of the following activities please tick the degree of **difficulty** you have experienced during the **PAST 4 WEEKS** due to the hip **that you had replaced**. Please tick (3) one box only.

**What degree of difficulty do you have with…**

None ^1^ Mild ^2^ Moderate ^3^ Severe ^4^ Extreme ^5^

1. …descending stairs?

2. …ascending stairs?

3. …rising from sitting?

4. …standing?

5. …bending to floor?

6. …walking on flat?

7. …getting in/out of car?

8. …going shopping?

9. …putting on socks/stockings?

10. …rising from bed?

11. …taking off socks/stockings?

12. …lying in bed?

13. …getting in/out of bath/shower?

14. …sitting?

15. …getting on/off toilet?

16. …heavy household chores?

17. …light household chores?

The following questions concern the amount of hip **stiffness** (not pain) you have experienced during the **PAST 4 WEEKS** in your hip that you had replaced. Stiffness is a sensation of restriction or slowness in the ease with which you move your joints.

How much stiffness do you have after…

None ^1^ Mild ^2^ Moderate ^3^ Severe ^4^ Extreme ^5^

18. …first waking in the morning?

19. …sitting, lying or resting later in the day?

Bellamy N, Buchanan WW, Goldsmith CH, Campbell J, Stitt LW. Validation study of WOMAC: a health status instrument for measuring clinically important patient relevant outcomes to antirheumatic drug therapy in patients with osteoarthritis of the hip or knee. J Rheumatol 1988; 15(12): 1833-40.

##### EQ5D-3L Health Questionnaire

By placing a tick (3) in one box in each group below, please indicate which statements best describe your own health state **TODAY**. Please tick (3) one box only.a. Mobility

I have no problems in walking about ^1^

I have some problems in walking about ^2^

I am confined to bed ^3^b. Self-Care

I have no problems with self-care ^1^

I have some problems washing or dressing myself ^2^

I am unable to wash or dress myself ^3^

c. Usual Activities (e.g. work, study, housework, family or leisure activities)

I have no problems with performing my usual activities^1^ I have some problems with performing my usual activities ^2^

I am unable to perform my usual activities ^3^d. Pain/Discomfort

I have no pain or discomfort ^1^

I have moderate pain or discomfort ^2^

I have extreme pain or discomfort ^3^e. Anxiety/Depression

I am not anxious or depressed ^1^

I am moderately anxious or depressed ^2^

I am extremely anxious or depressed ^3^

EuroQol—a new facility for the measurement of health-related quality of life. Health Policy 1990; 16(3): 199-208.

##### Self-Administered Patient Satisfaction Scale

The following questions concern your satisfaction with aspects of your hip replacement. For each question, please tick the box that best represents how satisfied you are with your hip replacement.

1. How satisfied are you with the results of your surgery?

2. How satisfied are you with the results of your surgery for improving your pain?

3. How satisfied are you with the results of surgery for improving your ability to do home or yard work?

4. How satisfied are you with the results of surgery for improving your ability to do recreational activities?

Very satisfied ^1^

Somewhat satisfied ^2^

Somewhat dissatisfied ^3^

Very dissatisfied ^4^

Mahomed N, Gandhi R, Daltroy L, Katz J. The Self-Administered Patient Satisfaction Scale for Primary Hip and Knee Arthroplasty. Arthritis 2011; Article ID 591253.

^Oxford Hip Score^

During the past 4 weeks

1.How would you describe the pain you usually have in your hip?

None ^1^

Very mild ^2^

Mild ^3^

Moderate ^4^

Severe ^5^

2.Have you been troubled by pain from your hip in bed at night?

No nights ^1^

Only 1 or 2 nights ^2^

Some nights 3

Most nights ^4^

Every night ^5^

3.Have you had any sudden, severe pain (shooting, stabbing, or spasm) from your affected hip?

No days ^1^

Only 1 or 2 days ^2^

Some days ^3^

Most days ^4^

Every day ^5^

4.Have you been limping when walking because of your hip?

Rarely/never ^1^

Sometimes or just at first ^2^

Often, not just at first ^3^

Most of the time ^4^

All of the time ^5^

5.For how long have you been able to walk before the pain in your hip becomes severe (with or without a walking aid)?

No pain for 30 minutes or more ^1^

16 to 30 minutes ^2^

5 to 15 minutes ^3^

Around the house only ^4^

Not at all ^5^

6. Have you been able to climb a flight of stairs?

Yes, easily ^1^

With little difficulty ^2^

With moderate difficulty ^3^

With extreme difficulty ^4^

No, impossible ^5^

7.Have you been able to put on a pair of socks, stockings or tights?

Yes, easily ^1^

With little difficulty ^2^

With moderate difficulty ^3^

With extreme difficulty ^4^

No, impossible ^5^

8.After a meal (sat at a table), how painful has it been for you to stand up from a chair because of your hip?

Not at all painful ^1^

Slightly painful ^2^

Moderately painful ^3^

Very painful ^4^

Unbearable ^5^

9.Have you had any trouble getting in and out of a car or using public transportation because of your hip?

No trouble at all ^1^

Very little trouble ^2^

Moderate trouble ^3^

Extreme difficulty ^4^

Impossible to do ^5^

10.Have you had any trouble washing and drying yourself (all over) because of your hip?

No trouble at all ^1^

Very little trouble ^2^

Moderate trouble ^3^

Extreme difficulty ^4^

Impossible to do ^5^

11.Could you do the household shopping on your own?

No trouble at all ^1^

Very little trouble ^2^

Moderate trouble ^3^

Extreme difficulty ^4^

Impossible to do ^5^

12.How much has pain from your hip interfered with your usual work, including housework?

Not at all ^1^

A little bit ^2^

Moderately ^3^

Greatly ^4^

Totally ^5^

Dawson J, Fitzpatrick R, Carr A, Murray D. Questionnaire on the perceptions of patients about total hip replacement. J Bone Joint Surg (Br) 1996; 78-B(2): 185-90.
