# Supplementary material for: Validated repeatability of patient-reported outcome measures following primary total hip replacement: a mode of delivery comparison study with randomized sequencing
Source: Acta Orthop. 2018 Nov 19;89(6):628–33. doi: 10.1080/17453674.2018.1521183 (PMC6300745; doi:10.1080/17453674.2018.1521183)
Supplement: Supplemental Material [file IORT_A_1521183_SM3668.pdf]

Supplementary data

Table 2. Missing data. Values are frequency

	Face-to-face			Self-administered			Telephone			Postal		
	Pts	Items	Excluded	Pts	Items	Excluded	Pts	Items	Excluded	Pts	Items	Excluded
WOMAC stiffness	0	0	0	0	0	0	1	1	0	1	2	1
WOMAC pain	0	0	0	0	0	0	0	0	0	0	0	0
WOMAC function	2	6	1	2	6	1	4	5	0	3	6	0
OHS	2	13	1	4	23	3	1	1	0	1	12	1
EQ5D	0	0	0	0	0	0	0	0	0	1	1	1
SAPS	0	0	0	0	0	0	0	0	0	0	0	0

Pts: participants

Table 3. Mean differences by mode. Values are mean (standard deviation)

PROM	Face-to-face	Self-administered	Postal	Telephone	F test
WOMAC stiffness	15.9 (22)	15.5 (22)	13.3 (16)	9.1 (15)	P F (3, 259) = 0.2
WOMAC pain	12.1 (17)	12.1 (17)	10.2 (16)	10.1 (16)	P F (3, 260) = 0.8
WOMAC function	15.4 (19)	15.4 (19)	14.1 (18)	12.1 (16)	P F (3, 260) = 0.7
OHS	39.8 (10)	40.2 (10)	40.1 (9.8)	42.0 (7.7)	P F (3, 258) = 0.6
EQ5D index	0.74 (0.30)	0.74 (0.30)	0.77 (0.30)	0.77 (0.24)	P F(3, 259) = 0.8
SAPS	93.4 (8.7)	89.2 (16)	93.6 (14)	94.3 (13)	P F (3, 260) = 0.09

Appendix

PROMs questionnaires

The WOMAC is a 24-item questionnaire designed to measure pain, function, and stiffness in hips and knees. It uses a 5-point Likert-type scale from 0 to 4 for each question (giving a total scale of 0–96) with higher scores indicating worse outcomes. The minimal clinically important difference (MCID) for the WOMAC is 15 for stiffness, 23 for pain, and 19 for function (Quintana et al. 2005). A percentage score for each subscale of the WOMAC was calculated, giving a score out of 100 for stiffness, pain, and function. EQ5D-3L is a standardized, non-disease-specific questionnaire for evaluating health-related quality of life in 5 dimensions, including mobility, self-care, usual activities, pain or discomfort, and anxiety or depression. The EQ5D index is calculated to provide a single value for health status, using the user written EQ5D Stata command in Stata. SAPS is a short questionnaire used to evaluate patient satisfaction with total hip and knee replacement. 4 items are scored on a 4-point Likert scale with responses from very dissatisfied to very satisfied. The scale score is the unweighted mean of the scores, with 100 being most satisfied and 25 least. The OHS is a 12-item questionnaire designed to measure changes in pain and function after total hip replacement surgery. The OHS is scored on a 0–48 scale, where 48 represents a good hip and 0 the worst (Murray et al. 2007). The MCID for the OHS is 5 (Beard et al. 2015).

The sets of PROMs were delivered by 4 modes: self-administered in clinic and face-to-face interviewer led, both completed during the outpatient clinic appointment; and later via telephone interview and self-administered by post. Participants completing PROMs self-administered in clinic were asked to complete the set of questionnaires using pen and paper, without assistance. During the face-to-face interview, each question in the set was asked by a member of the research team and the questionnaires were completed by the researcher based on the verbal responses of the participant.

Western Ontario and McMaster Universities Osteoarthritis Index

The following questions concern the amount of pain you have recently experienced in your hip. For each question, please tick the amount of **pain** you have experienced during the **PAST 4 WEEKS** due to the hip **that you had replaced**. Please (✓) tick one column.

1. How much pain do you have **walking on a flat surface**?
   None 1 Mild 2 Moderate 3 Severe 4 Extreme 5
2. How much pain do you have **going up or down stairs**?
   None 1 Mild 2 Moderate 3 Severe 4 Extreme 5
3. How much pain do you have **at night while in bed**?
   None 1 Mild 2 Moderate 3 Severe 4 Extreme 5
4. How much pain do you have **sitting or lying**?
   None 1 Mild 2 Moderate 3 Severe 4 Extreme 5
5. How much pain do you have **standing upright**?
   None 1 Mild 2 Moderate 3 Severe 4 Extreme 5

The following questions concern your physical function. By this we mean your ability to move around and look after yourself. For each of the following activities please tick the degree of **difficulty** you have experienced during the **PAST 4 WEEKS** due to the hip **that you had replaced**. Please tick (✓) one box only.

What degree of difficulty do you have with...

None 1 Mild 2 Moderate 3 Severe 4 Extreme 5

1. ...descending stairs?
2. ...ascending stairs?
3. ...rising from sitting?
4. ...standing?
5. ...bending to floor?
6. ...walking on flat?
7. ...getting in/out of car?
8. ...going shopping?
9. ...putting on socks/stockings?
10. ...rising from bed?
11. ...taking off socks/stockings?
12. ...lying in bed?
13. ...getting in/out of bath/shower?
14. ...sitting?
15. ...getting on/off toilet?
16. ...heavy household chores?
17. ...light household chores?

The following questions concern the amount of hip **stiffness** (not pain) you have experienced during the **PAST 4 WEEKS** in your hip that you had replaced. Stiffness is a sensation of restriction or slowness in the ease with which you move your joints.

How much stiffness do you have after…

None 1 Mild 2 Moderate 3 Severe 4 Extreme 5

18. …first waking in the morning?
19. …sitting, lying or resting later in the day?

Bellamy N, Buchanan WW, Goldsmith CH, Campbell J, Stitt LW. Validation study of WOMAC: a health status instrument for measuring clinically important patient relevant outcomes to antirheumatic drug therapy in patients with osteoarthritis of the hip or knee. J Rheumatol 1988; 15(12): 1833-40.

EQ5D-3L Health Questionnaire

By placing a tick (✓) in one box in each group below, please indicate which statements best describe your own health state **TODAY**. Please tick (✓) one box only.

a. Mobility
   I have no problems in walking about 1
   I have some problems in walking about 2
   I am confined to bed 3
b. Self-Care
   I have no problems with self-care 1
   I have some problems washing or dressing myself 2
   I am unable to wash or dress myself 3
c. Usual Activities (e.g. work, study, housework, family or leisure activities)
   I have no problems with performing my usual activities 1
   I have some problems with performing my usual activities 2
   I am unable to perform my usual activities 3
d. Pain/Discomfort
   I have no pain or discomfort 1
   I have moderate pain or discomfort 2
   I have extreme pain or discomfort 3
e. Anxiety/Depression
   I am not anxious or depressed 1
   I am moderately anxious or depressed 2
   I am extremely anxious or depressed 3

EuroQol—a new facility for the measurement of health-related quality of life. Health Policy 1990; 16(3): 199-208.

Self-Administered Patient Satisfaction Scale

The following questions concern your satisfaction with aspects of your hip replacement. For each question, please tick the box that best represents how satisfied you are with your hip replacement.

1. How satisfied are you with the results of your surgery?
2. How satisfied are you with the results of your surgery for improving your pain?
3. How satisfied are you with the results of surgery for improving your ability to do home or yard work?
4. How satisfied are you with the results of surgery for improving your ability to do recreational activities?

Very satisfied 1
Somewhat satisfied 2
Somewhat dissatisfied 3
Very dissatisfied 4

Mahomed N, Gandhi R, Daltroy L, Katz J. The Self-Administered Patient Satisfaction Scale for Primary Hip and Knee Arthroplasty. Arthritis 2011; Article ID 591253.

Oxford Hip Score

During the past 4 weeks

1. How would you describe the pain you usually have in your hip?
   None 1
   Very mild 2
   Mild 3
   Moderate 4
   Severe 5

2. Have you been troubled by pain from your hip in bed at night?
   No nights 1
   Only 1 or 2 nights 2
   Some nights 3
   Most nights 4
   Every night 5

3. Have you had any sudden, severe pain (shooting, stabbing, or spasm) from your affected hip?
   No days 1
   Only 1 or 2 days 2
   Some days 3
   Most days 4
   Every day 5

4. Have you been limping when walking because of your hip?
   Rarely/never 1
   Sometimes or just at first 2
   Often, not just at first 3
   Most of the time 4
   All of the time 5

5. For how long have you been able to walk before the pain in your hip becomes severe (with or without a walking aid)?
   No pain for 30 minutes or more 1
   16 to 30 minutes 2
   5 to 15 minutes 3
   Around the house only 4
   Not at all 5

6. Have you been able to climb a flight of stairs?
   Yes, easily 1
   With little difficulty 2
   With moderate difficulty 3
   With extreme difficulty 4
   No, impossible 5

7. Have you been able to put on a pair of socks, stockings or tights?
   Yes, easily 1
   With little difficulty 2
   With moderate difficulty 3
   With extreme difficulty 4
   No, impossible 5

8. After a meal (sat at a table), how painful has it been for you to stand up from a chair because of your hip?
   Not at all painful 1
   Slightly painful 2
   Moderately painful 3
   Very painful 4
   Unbearable 5

9. Have you had any trouble getting in and out of a car or using public transportation because of your hip?
   No trouble at all 1
   Very little trouble 2
   Moderate trouble 3
   Extreme difficulty 4
   Impossible to do 5

10. Have you had any trouble washing and drying yourself (all over) because of your hip?
    No trouble at all 1
    Very little trouble 2
    Moderate trouble 3
    Extreme difficulty 4
    Impossible to do 5

11. Could you do the household shopping on your own?
    No trouble at all 1
    Very little trouble 2
    Moderate trouble 3
    Extreme difficulty 4
    Impossible to do 5

12. How much has pain from your hip interfered with your usual work, including housework?
    Not at all 1
    A little bit 2
    Moderately 3
    Greatly 4
    Totally 5

Dawson J, Fitzpatrick R, Carr A, Murray D. Questionnaire on the perceptions of patients about total hip replacement. J Bone Joint Surg (Br) 1996; 78-B(2): 185-90.
